# Joel G. Breman, ASTMH Past President (1936–2024)

**DOI:** 10.4269/ajtmh.24-0626

**Published:** 2024-10-29

**Authors:** Richard W. Steketee, Philip J. Rosenthal

**Affiliations:** ^1^ Independant consultant, Bethesda, Maryland;; ^2^ Department of Medicine, University of California–San Francisco, San Francisco, California

Joel G. Breman, MD, DTPH, FIDSA, FASTMH, former president of the American Society of Tropical Medicine and Hygiene (ASTMH) and a public health giant of the late twentieth and early twenty-first centuries, died in April of 2024 at age 87 after a long illness. Obituaries have already been published,^1^^-^^6^ but we wish to briefly summarize the remarkable impact that our friend Joel had on international public health, on the ASTMH, and on us.

One of us (R.W. Steketee), first met Joel in 1985 when joining the Centers for Disease Control and Prevention (CDC) Malaria Branch and then worked directly under Joel’s supervision for 7 years. Another of us (P.J. Rosenthal), met Joel in the 1990s, when Joel was a leader at the Fogarty International Center. Joel recommended Burkina Faso as a research site, where he had worked early in his career, and provided a key contact which led to a productive collaboration in that country that continues to this day. These interactions, both involving work on malaria, came well after Joel had established himself as an influential force in studying other tropical diseases.

The number and diversity of diseases that Joel tackled was truly remarkable. Early in his career, he controlled a large outbreak of botulism (associated with ingestion of Mexican hot sauce) in Michigan. Given his experience and skills, CDC moved him to west Africa in the late 1960s to help in the eradication of smallpox. In 1976, in the midst of helping to rid the world of smallpox, he found himself in rural Zaire investigating an unknown but newly recognized outbreak of hemorrhagic fever with very high fatality – the disease that we know today as Ebola. Joel later described this as “the scariest epidemic of my entire medical career,” as he and others worked to characterize a severe outbreak before its biology or means of transmission was understood. These efforts were highlighted in *The Hot Zone*, by Richard Preston. With Joel’s smarts and knack for collaboration, CDC moved him to Geneva to be the deputy Chief of the WHO smallpox unit, where he participated in much of the work that led to the certification of smallpox eradication in 1980. Not bad for a first decade in public health – playing critical roles in the eradication of one devastating viral infection and the discovery of another.

**Figure:**
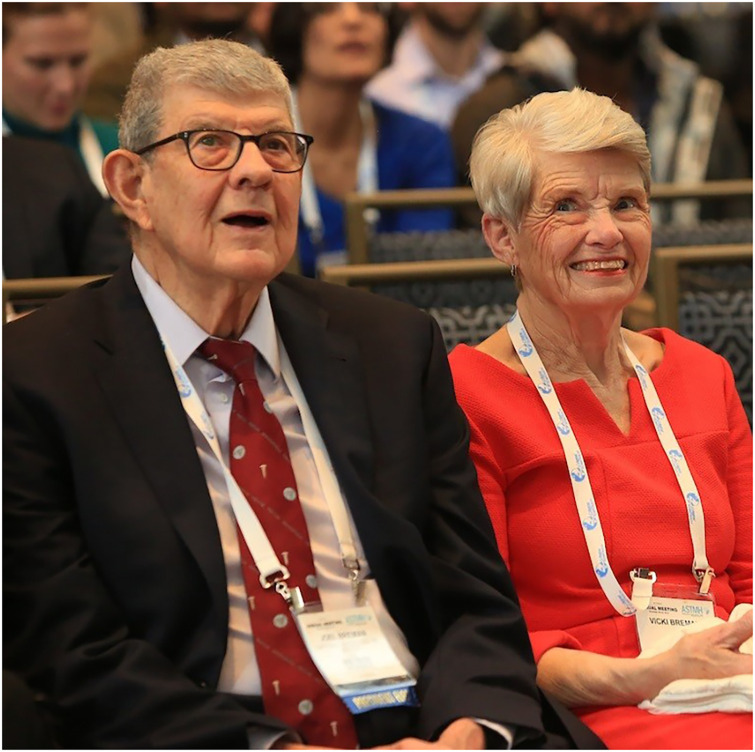
Joel G. Breman, ASTMH Past President

Joel returned to CDC in Atlanta and took a position in the revived Malaria Branch, where he served as the deputy Chief responsible for epidemiology and field work. He mentored many young scientists (including R.W. Steketee) in the exploration of parasite, vector, human, and health-system aspects of malaria. In 1995, Joel joined the Fogarty International Center, the branch of the NIH that specifically focuses on facilitating training and research on global health. Joel served there for 15 years, bringing insights and enthusiasm to the international portfolio of research and training of the Center. In 2020, late in his career and already in his 80s, Joel served as President of the ASTMH. Joel brought to ASTMH his enthusiasm for research, training, and advocacy; he was a particularly strong supporter of this Journal. His experience and wisdom proved highly valuable during the difficult early days of the COVID-19 pandemic. As stated by Karen Goraleski, the ASTMH CEO at that time, “As the pandemic unfolded, he was the right President at the right time. He had a clear understanding of the seriousness of the pandemic, and without any naivete, radiated calm and assurances that we would weather this global calamity. We are all a little better off for being in Joel’s expansive orbit.”

While Joel was an expert in infectious diseases and their clinical, epidemiologic, and microbiologic features, his greatest passion and talent was focused on building health systems to reach the disenfranchised. He was a patient and devoted teacher, and he loved interacting with enthusiastic young people with fresh ideas. As a leader, he never micromanaged but offered support that encouraged motivation in his colleagues. Joel recognized that his contributions were always part of a team effort, and he took the greatest pride in reducing the impact of disease, rather than in personal fame or recognition. His own history with disease eradication and new disease detection gave him a unique perspective and allowed him to bring that perspective to realistic enthusiasm and hope for malaria elimination, as well as for eradication of other infectious diseases, including Guinea worm, onchocerciasis, polio, and measles.

Joel loved life and cared about people – his family, his professional colleagues, and those disenfranchised and in need – especially young children and others in Africa. Collaboration was his compass, and his work in public health programs and research involved colleagues around the world. He was always eager to listen and learn from each person he met, regardless of their status or role. He was equally comfortable speaking with an African Minister of Health, a global leader on a specific disease, a class of public health students, a bus driver, or a custodian in his office building.

Amidst his very busy professional life, Joel was always a loving, devoted husband, father and grandfather. Somehow, he found time for travel, meeting new people, and sharing stories – lots of stories. Joel had a hearty laugh, loved full moons, colorful clothing, licorice, and all varieties of music. He proudly coached his son’s soccer teams, played the clarinet, enjoyed hiking, ski trips with his family, rowing on the Potomac River, biking through the US and Europe, and jogging until his early 80s. He loved vacations with his children and grandchildren, including memorable trips to Brazil, where he celebrated his 70th birthday dancing down the streets during Carnaval. He even climbed the summit of Mt. Kilimanjaro in East Africa with his daughter.

Joel’s contributions in public health were as diverse as they were impactful – playing a key role in the successful eradication of smallpox; working toward eradication of other infections, many of which have seen great progress in recent years; characterizing Ebola soon after its discovery; working to control and eventually eliminate malaria in Africa; and recently, addressing COVID-19 in underserved communities and the emergence of mpox. By purely academic standards he was remarkably successful, with nearly 140 papers (published from 1970 to 2023) in influential journals, textbooks, and book chapters that guide medicine and public health practice, and in particular the practice of infectious disease control and elimination, especially in low resource countries. And more is coming: his book *The Principles and Practice of Disease Eradication* (with Jon Andrus) – a fantastic culmination of much of Joel’s life work, will be published in late 2024. Donations in remembrance of Joel can be directed to the Breman Global Health Immersion Fellowship at the University of Southern California, where he received his MD in 1965. Over his long career, Joel’s impact on so many people in so many places was vast. He is greatly missed, but his impact lives on.
